# Solid organ fabrication: comparison of decellularization to 3D bioprinting

**DOI:** 10.1186/s40824-016-0074-2

**Published:** 2016-08-31

**Authors:** Jangwook P. Jung, Didarul B. Bhuiyan, Brenda M. Ogle

**Affiliations:** 1 Department of Biomedical Engineering, University of Minnesota – Twin Cities, 312 Church St. SE, Minneapolis, MN 55455 USA; 2 Stem Cell Institute, University of Minnesota – Twin Cities, 312 Church St. SE, Minneapolis, MN 55455 USA; 3 Masonic Cancer Center, University of Minnesota – Twin Cities, 312 Church St. SE, Minneapolis, MN 55455 USA; 4 Lillehei Heart Institute, University of Minnesota – Twin Cities, 312 Church St. SE, Minneapolis, MN 55455 USA; 5 Institute for Engineering in Medicine, University of Minnesota – Twin Cities, 312 Church St. SE, Minneapolis, MN 55455 USA

**Keywords:** Extracellular matrix, 3D printing, Decellularization, Organogenesis, Biomimetics

## Abstract

Solid organ fabrication is an ultimate goal of Regenerative Medicine. Since the introduction of Tissue Engineering in 1993, functional biomaterials, stem cells, tunable microenvironments, and high-resolution imaging technologies have significantly advanced efforts to regenerate in vitro culture or tissue platforms. Relatively simple flat or tubular organs are already in (pre)clinical trials and a few commercial products are in market. The road to more complex, high demand, solid organs including heart, kidney and lung will require substantive technical advancement. Here, we consider two emerging technologies for solid organ fabrication. One is decellularization of cadaveric organs followed by repopulation with terminally differentiated or progenitor cells. The other is 3D bioprinting to deposit cell-laden bio-inks to attain complex tissue architecture. We reviewed the development and evolution of the two technologies and evaluated relative strengths needed to produce solid organs, with special emphasis on the heart and other tissues of the cardiovascular system.

## Background

Tissue Engineering, as introduced in 1993 [1], is the creation of complex tissues and organs from simpler engineered pieces. Over the past few decades, biomaterials, stem cell technology and advanced imaging modalities have been developed to generate tissue components. Biomaterials are not only a delivery vehicle or passive scaffold for cells, but dynamically modulated microenvironments in vivo and in vitro. Synthetic biomaterials possess well-defined physicochemical properties and synthetic-natural hybrid biomaterials often present tunable biological functionalities. Stem cell biology with reprogramming and gene-editing provides more options for cell sources in tissue engineered constructs. Advanced imaging techniques enable acquisition of more detailed spatiotemporal information, which can serve as a blue print for tissue regeneration [2, 3].

**Fig. 1:**
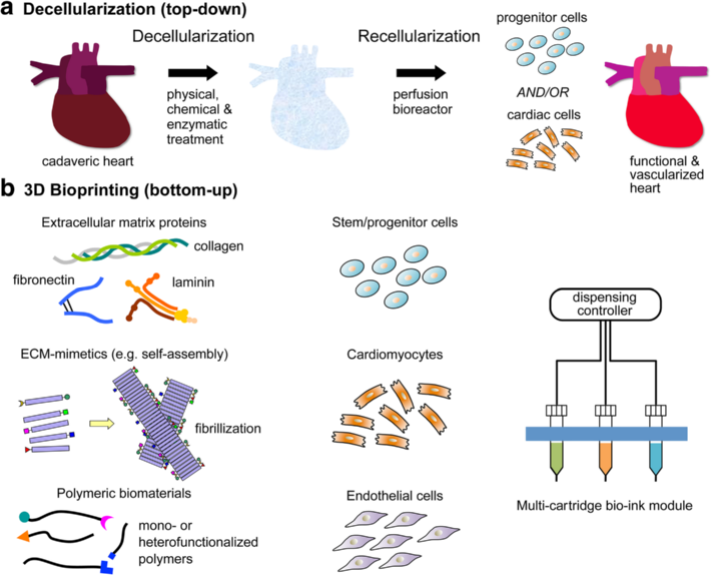
Two representative regenerative medicine technologies for cardiac organogenesis. **a** Decellularization starts with physical, chemical, and enzymatic treatment of cadaveric heart to remove cells while keeping the extracellular matrix of the heart. Utilizing a perfusion bioreactor, the decellularized heart is repopulated with either progenitor cells or terminally differentiated cardiac cell types. The end goal of this strategy is to regenerate functional heart with complete vascularization. **b** Functional biomaterials (ECM proteins, ECM-analogues or synthetic materials) and/or cells (stem, progenitor or fully differentiated) serve as a bio-ink for 3D bioprinting. This controlled manufacturing technology aims to produce spatially-defined tissues or organs at multiple length scales

Earlier approaches in Tissue Engineering focused on 2D organs such as skin and hollow tubular (e.g. blood vessels) or non-tubular hollow organs (e.g. bladder). Solid organs such as kidney, liver or heart are the most complex in achieving vascularization and innervation [4]. Thus solid organs are more than a collection of 2D tissue components and need to be created by exploiting all-in-one approaches from the beginning. Generating simple 2D or hollow organs is feasible with cell and supporting scaffold of only one type by molding them into a pre-designed cast. However, molding-based fabrication is challenging to accommodate multiple cell types and the extracellular matrix (ECM) in 3D space to achieve tissue-mimicking patterns and associated spatial resolution. Fortunately two technologies have recently emerged that are likely to facilitate solid organ fabrication ex vivo, namely decellularized tissue scaffolds and 3D bioprinting. These fabrication technologies are quite distinct in their execution (Fig. 1), and therefore harbor distinct attributes and limitations. In this review, we will describe decellularization and 3D bioprinting for soft tissue regeneration in detail, and briefly summarize the pros and cons of each especially in the context of the cardiovascular system. We also comment on the merging of these technologies via 3D printing with decellularized ECM bio-ink and discuss biomimetic 4D printing. Readers are referred to other reviews for specific, emerging technical advances that enable 3D bioprinting (Murphy and Atala [4], O’Brien et al. [5], Studart [6], Jungst et al. [7], Jose et al. [8], Guvendiren et al. [9] and Gudapati et al. [10]).

### Organogenesis via decellularization and recellularization

**Fig. 2:**
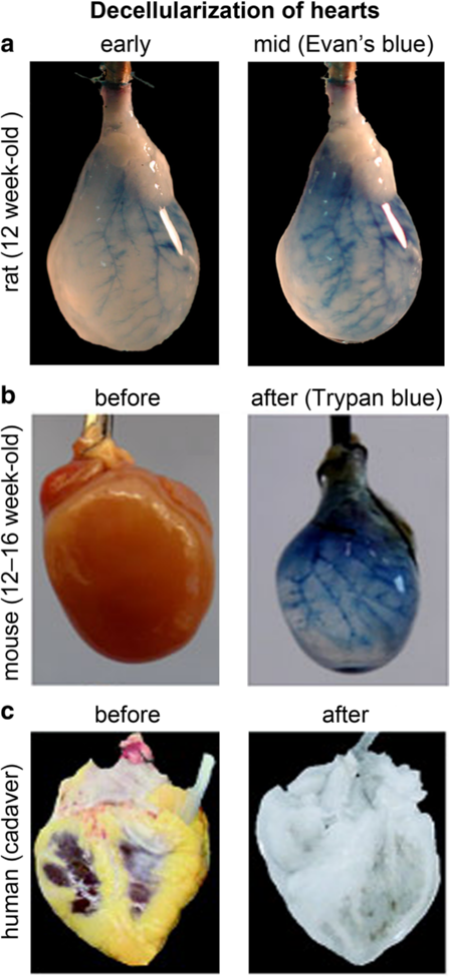
Decellularization of mammalian hearts. **a** Decellularization of rat heart with 1 % SDS, 1 % Triton X-100, and antibiotic-containing PBS. Macroscopic view of Evan’s blue dye perfusion showing intact coronary vasculature, with permission from Nature Publishing Group [13]. **b** Decellularization of mouse heart with trypsin, 1 % SDS, 3 % Triton X-100, and 0.1 % peracetic acid, with permission from Nature Publishing Group [20]. The intact coronary vasculature visualized with Trypan blue solution. **c** Cadaveric human heart before and after perfusion decellularization, with permission from Elsevier [14]

A number of approaches have been attempted to remove cells and preserve intact ECM via physical, chemical and enzymatic treatments [11]. Decellularized scaffolds provide architecture and mechanical integrity of the remaining ECM, while avoiding adverse biological and immunological responses from cellular and nuclear materials. One of the earliest approaches was to harvest the ECM of the small intestinal submucosa (SIS) by removing the superficial layers of the mucosa and the external muscular layers [12]. Although this was suitable for 3D in vitro cell cultures, 80 μm-thick ECMs may not have mechanical properties to resist shear from cardiac pumping and circulation. Expanding this idea to organ scale, the first decellularized heart was described by Ott et al. [13] in 2008. This pioneering approach reported the decellularization of 12 week-old whole rat heart while preserving the underlying ECM and intact geometry and producing acellular and perfusable vasculature (Fig. 2a). The decellularized rat heart was repopulated with rat aortic endothelial and neonatal cardiac cells and was matured further with physiological preload, afterload and intraventricular pressure as well as electrical stimulation at 5–20 V in a bioreactor up to 28 days. This recellularized heart developed macroscopic contraction and the measured pump function was equivalent to about 2 % of adult or 25 % of 16-week fetal heart function. This inaugural study initiated regenerative engineering approaches in different types of solid organs including lung, kidney and pancreas [14]. Since then, decellularization procedures have been further optimized and critically evaluated [15].

From an organogenesis perspective, decellularized tissues maintain the ECM framework, but lack the fundamental and biological unit of organs. Although the first human embryonic stem cells (ESC) were derived in 1998 [16], the idea of employing stem or progenitor cells became more attractive for tissue regeneration after the introduction of induced pluripotent stem cells (iPSCs) [17, 18]. This breakthrough stem cell technology might enable production of a personalized heart. Followed by decellularization, repopulating the decellularized heart with either pluripotent stem cells or lineage-restricted progenitor cells [19] and maturing those cells within the intact ECMs in situ could promote the generation of functional heart tissue. Lu et al. [20] decellularized mouse cadaveric whole heart (Fig. 2b) and repopulated with human iPS-derived multipotent cardiovascular progenitors (MCPs), which were differentiated in situ into cardiomyocytes, smooth muscle cells and endothelial cells. After 20 days of perfusion, the repopulated heart exhibited myocardium and vessel-like structures, contracted spontaneously at a rate of 40–50 beats per min and responded to isoproterenol treatment. This study also found that cardiac ECM prompted significantly more cardiomyocyte proliferation, differentiation and myofilament formation from the repopulated MCPs than in a 3D microenvironment of embryoid body. More recently, decellularization and repopulation with iPSCs was up-scaled to human cadaveric heart. Guyette et al. [21] prepared decellularized human cadaveric whole heart from 73 donors via perfusion decellularization (Fig. 2c). Toward better translational outcome, this group employed cardiomyocytes from a non-transgenic (RNA-induced) PSCs [22] by modulating the Wnt/β-catenin signaling with inhibitors of GSK3β and Wnt [23]. The whole heart recellularization was performed using a custom bioreactor capable of providing coronary perfusion and left ventricular wall mechanical stimulation that was achieved by oscillating the pressure inside a balloon placed inside the left ventricle. Heart is not the only candidate organ for decellularization and repopulation with progenitors or terminally differentiated cells. These approaches were also applied to liver [24], kidney [25] and other cardiovascular tissues including valves and vessels [26] (authors refer to a recent review by Song and Ott [14] for further review of decellularizaton of others organs).

Preserving vasculature and mechanical integrity of the tissue with decellularization allows repopulation of the decellularized organs with cells. For clinical application, techniques to achieve complete endothelialization with recellularization will be critical for the maintenance of blood flow and the prevention of blood coagulation given the thrombogenicity of certain ECM proteins of the decellularized scaffold [27].

In summary, decellularization of whole organ has been achieved with intact ECM architecture and perfusable vasculature. Preserving vasculature and mechanical integrity of the tissue with decellularization allows repopulation of the decellularized organs with cells. Repopulation with iPSCs have been achieved up to human scale for certain organs. However, the number of different types of cells for repopulation of decellularized organs is limited. In particular, the spatial acuity or precise positioning of cells is challenging to achieve as repopulation is currently dependent on perfusion. Recently emerged 3D bioprinting technique has the potential to fulfill some of these limitations and complement the advantages of decellularization-based regenerative engineering.

### 3D bioprinting for directed morphogenesis and organogenesis

3D printing has been a disruptive innovation in many areas of industry, research, medicine and education. 2D printing technology of materials has advanced to an additive process in which successive layers are formed into a 3D shape. Soon after 3D printing, termed stereolithography, was first introduced in 1986 by Charles W. Hull, it began to replace many manufacturing and molding processes. Since medical devices or prosthetics need to be produced with customized detail in clinics, 3D printing is an ideal candidate to augment personalized medicine applications. Recently, combining high-resolution imaging, computer-aided design and resorbable biomaterials, 3D printing showcased an airway splint to treat an infant with tracheobronchomalacia [28]. Later, further advancements were made to include multiple types of precisely printable biological ink (bio-ink), sacrificial biomaterials, and living cells to fabricate bone, cartilage and skeletal muscle [29]. In this section, we will discuss the advancement of 3D printing via biomaterials and living cells with an emphasis on cardiovascular tissues, followed by hybrid approaches such as 3D bioprinting with decellularized ECM for complex, soft tissues.

#### Transition from 2D patterning to 3D bioprinting

3D bioprinting began as 2D patterning with biomaterials with cells or multilayered 3D cell-laden hydrogels structures (for example, smooth muscle cells (SMCs) encapsulated in collagen type I with 16.2 μm thick layers [30]. One of the earliest attempts was to employ a Hewlett-Packard Desktop printer (HP 550C) and a modified HP 51626a ink cartridge to print Chinese Hamster Ovary (CHO) cells [31]. These cell suspensions (bio-inks) were printed as a circular pattern on two different types of hydrogel-based substrates (bio-paper) that were prepared from either soy agar or collagen type I. Only around 8 % of CHO cells were lysed during printing and maintained viability up to 25 days. As biomaterials serve as instructive cues for directing morphogenesis [32] and as protective matrices, bio-inks (cells and biomaterials) incapable of maintaining cell health would be detrimental to printed tissue or organs. One intriguing bio-ink approach incorporated tissue spheroids (homo and heterocellular cell aggregates) as building blocks, which were assembled into vascularized thick tissue constructs [33]. As one example, single- and double-layered small diameter vascular tubes ranging from outer diameter 0.9 to 2.5 mm were generated via layer-by-layer bioprinting and self-assembled multicellular (CHOs, SMCs and fibroblasts) spheroids with agarose cylinders as molding templates [34].

#### Complex 3D bioprinting of multiple cell types with multiple biomaterials

From aforementioned studies, 3D bioprinting technologies further evolved to provide more complex tissue structures by incorporating multiple biomaterials (bio-inks) to confer appropriate biochemical and mechanical properties to engineered organs. Since single material, single cell type, single stage processing and simple patterns cannot mimic the complex nature of tissues or organs, recent approaches have included multiple biomaterials with orthogonal chemistry and multiple differentiated cells or stem cells for in situ differentiation. Also, high-resolution image-guided patterns are now routinely printed with reasonable fidelity at several micro- and sub-micrometer scales for certain printing approaches [35].

Adapting the aforementioned inkjet printing technology, amniotic fluid derived stem cells (AFSCs), SMCs and endothelial cells (ECs) were printed onto a multi-cell pie configuration in an alginate and collagen composite in an attempt to mimic bone structure [36]. The printed composite exhibited similar potassium (K) current-voltage relationships at 7 day in vitro and 4 week in vivo, indicating the basic membrane electrical properties of SMCs were maintained after 3D bioprinting. After 7 days in culture and implantation, ECs in the printed composites showed no significant difference of bradykinin-induced intracellular Ca^2+^ concentration ([Ca^2+^]_i_), indicative of maintaining normal vasodilation properties. After 7 days of osteogenic induction and 18-week implantation, the printed composite expressed osteocalcin and formed bony structures with significantly higher compressive modulus. Gaetani et al. [37] attempted to deliver progenitor cells in a 3D-printed gelatin/hyaluronic acid (HA) patch to recover cardiac function in a mouse model of myocardial infarction (MI). Sca1^+^ cardiac progenitor cells (CPCs) were printed with the gelatin/HA composite to form a woodpile array 3D structure. Over 4 weeks in cardiomyogenic differentiation, the printed CPCs were differentiated to phenotypes expressing troponin I, cardiac actin, and connexin43. After 4 weeks of application to the MI area of the myocardium, the wall thickness was increased and the infarct fibrosis was decreased.

#### Complex tissue patterning via imaging technologies

As multiple cell types and multi-component bio-inks were explored for 3D bioprinting, image-guided patterning became feasible with high-resolution imaging technologies. With inkjet printing technologies, the complex blueprint of tissues has yet to be reproduced at single micrometer-scale resolution. Thus, complex tissue or vasculature patterns are challenging to transfer with high fidelity. Two-photon laser scanning lithography (TP-LSL) has been used to generate simple patterns with features as small as 1 μm in the lateral direction and 5 μm in the axial direction [35, 38]. TP-LSL was further developed to pattern vasculature of the cerebral cortex from confocal imaging [39]. TP-LSL patterning was able to mimic the neural and vascular components of the subependymal zone neural stem cell niche and neural progenitor cells were induced to express platelet/endothelial cell adhesion marker-1 (PECAM-1). More complex patterns of the microvasculature of the cerebral cortex were patterned with human umbilical vein endothelial cells (HUVECs) and mesenchymal progenitor cells (10 T1/2), forming tubular networks in 24 h of culture. Liver functions are tightly linked to the 3D microenvironments of hepatocytes with supporting endothelial and mesenchymal cells in a hexagonal lobule unit. Ma et al. [40] fabricated liver lobule and vascular structures with two-step digital light processing (DLP)-based 3D printing technology utilizing photocrosslinkable gelatin and HA to encapsulate hiPSC-derived hepatic cells and supporting cells (HUVECs and adipose-derived stem cells (ADSC)). The 3D triculture model showed greater hepatocyte spheroid formation by reorganizing them in the designated patterns and significantly higher expression of key enzymes (cytochrome P450) in liver drug metabolism, compared to 3D single hiPSC-derived progenitor cell models.

**Fig. 3:**
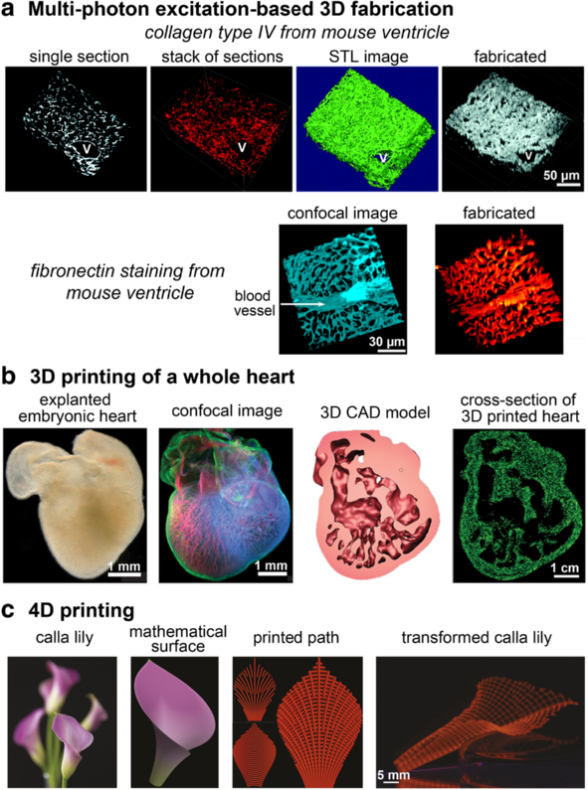
3D bioprinting of complex solid organs and biomimetic 4D printing. **a** A single section showing collagen type IV immunofluorescence staining in a postnatal day 2 ventricle. The stack of images was used to create a 3D reconstruction of the collagen type IV labeled serial sections and a solid stereolithography rendering was generated from the stack of 20 serial sections. Multi-photon excitation-based fabrication was then used to create a 3D construction, V indicates blood vessel [3]. Confocal images of fibronectin immunofluorescence staining from mouse ventricle and the fabricated structure created through modulated raster scanning, with permission from the Optical Society [35]. A blood vessel was indicated by the arrow. **b** An explanted embryonic chicken heart was stained for fibronectin (*green*), nuclei (*blue*), and F-actin (*red*). A cross-section of the fluorescence alginate (*green*) scaffold was printed from the 3D CAD model of the embryonic heart with the internal trabeculation utilizing the FRESH technology, with permission from the American Association of the Advancement of Science [46]. **c** 4D printing pathways [69]; mathematical surface was generated from natural inspiration and the path was printed with hydrogel composite ink of cellulose fibrils. Anisotropic swelling of the composite ink transformed the printed 2D paths into a 3D structure

Native tissue is a perfect bioinspired template to recreate 3D printed tissue structures. Mimicking developmental microenvironments can provide better signals for which stem or progenitor cells are originated and differentiated. Imaging based approaches can extract such spatial information [2]. Hanson et al. [3] acquired bioinspired templates from collagen architectures of developing heart using second harmonic generation (SHG) and spatial distribution of fibronectin, collagen type IV and elastin from embryonic day 12.5 (E12.5) to postnatal day 2 (P2) using a series of histologic sections stained for ECM proteins via immunofluorescence (Fig. 3a). 3D structures were printed based on such templates using multi-photon excitation (MPE)-based fabrication. The primary advantage of MPE-based 3D printing is sub-micrometer resolution to accommodate subtle and non-uniform ECM fibrillar structures with approximately 95 % fidelity as evidenced by scanning electron microscopy (SEM) [35]. Limiting MPE-based 3D printing is the inability to utilize bio-inks containing cells, though it is possible future iterations of this technology could accommodate such a feature [41].

#### 3D printing at the organ scale; important considerations

Once complex tissue structures are obtained, maintaining long-term cell survival and functionality is a key feature for regenerative medicine applications. For most tissues, this requires vascularization. Recently, Miller et al. [42] reported a 3D printed carbohydrate glass lattice to feed engineered organs, where the lattice structure is removed before introducing a homogenous cell-laden matrix. Primary rat hepatocytes showed significantly improved albumin secretion and urea synthesis in 3D printed vasculatures compared to a 3D slab lacking channels. To determine whether this 3D carbohydrate glass lattice can overcome the challenge of rapid oxygen and nutrient delivery to engineered vascularized tissue implanted in vivo, the 3D printed microvessel network was directly connected to rat femoral arteries [43]. The blood flow was similar to the positive control as evidenced by laser Doppler imaging at 1 and 3 h post-implantation. One limitation of the carbohydrate glass lattice approach is the need to print at elevated temperature (above 100 °C), which inhibits inclusion of cells in bio-inks. Kolesky et al. [44] developed an alternative approach to print 1D, 2D and 3D vasculatures with bio-inks including dermal fibroblasts/HUVECs and gelatin methacrylate at ambient temperature. To fabricate embedded vasculature, the cell-laden bio-ink contained fugitive ink composed of Pluronic F127 that can be easily printed and removed under mild conditions. This group recently reported a route for printing thicker vascularized tissues (>1 cm in thickness and 10 cm^3^ in volume) within a customized silicone-based perfusable chip for over 6 weeks [45]. The complex microenvironments were created first by printing hMSC-laden ink into a 3D lattice geometry along with intervening in- and out-of-plane features composed of fugitive ink, which transformed into a vascular network lined with HUVECs. After printing, the remaining interstitial space was filled with human neonatal dermal fibroblast (HNDF)-laden gelatin-fibrin mix, where hMSCs and HNDFs migrated toward the vascular channels.

Complex patterns and heterogeneous cell-laden structures have further evolved to organ level fabrication. One example, termed freeform reversible embedding of suspended hydrogels (FRESH) [46] demonstrated the ability to print complex anatomical architecture (Fig. 3b). After depositing a hydrogel precursor ink within a thermoreversible support bath (gelatin microparticles), in situ gelling was initiated at ambient conditions and then, the printed structure was heated to 37 °C to melt away the gelatin support bath. Using the FRESH printing approach, CT (computed tomography) or MRI (magnetic resonance imaging) data from bifurcated coronary arteries, femur, trabeculated embryonic heart, or human brain can be printed up to several millimeter scales at a resolution of around 200 μm from computer aided design (CAD) files. Another example demonstrated that human-scale tissue constructs could be printed using cell-laden hydrogels to fabricate bone, cartilage, and muscle tissues. Kang et al. [29] acquired medical imaging (CT or MRI) data and processed them in CAD software, translated to a series of command list for 3D printer XYZ stage movement and actuating pneumatic pressure and printed composite hydrogels for cell delivery including gelatin, fibrinogen, HA and glycerol mixed into high glucose DMEM (Dulbecco’s Modified Eagle Medium). This integrated tissue-organ printer (ITOP) was capable of printing at a resolution of 2 μm for biomaterials and around 50 μm for cells. Simultaneous printing of an outer sacrificial hydrogel that is dissolved later imparted mechanical strength to maintain the shape of the printed structures while creating an array of microchannels permissive to nutrient and oxygen diffusion into the printed structures. By modulating the patterns of the load-bearing poly(ε-caprolactone, PCL) with respect to other cell-laden bio-inks or sacrificial materials (Pluronic F-127), the mechanical properties could be tuned for different tissue or organ applications. For example, the authors printed stiffer structures that were for mandible bone or ear-shaped cartilage while more porous structures were employed to print skeletal muscle constructs.

#### 3D printing with decellularized ECM bio-ink

To fabricate solid organs, we have discussed approaches to utilize the ECM from decellularized organs (primarily heart) or to print cell-laden bio-inks consisting of multiple ECM proteins and cells (Fig. 1). In addition to these extracted and purified ECM proteins, decellularized ECM (dECM) has been processed and self-assembled as an injectable biomaterial for tissue recovery [47, 48] or cell culture templates to maintain more mature phenotypes in vitro [49]. Pati et al. [50] harvested multiple types of tissues to prepare dECM bio-ink and printed cell-laden dECM bio-inks with a PCL framework, where this printing technology was capable of printing feature sizes either at 100 or 200 μm. This multi-head tissue/organ building system (termed MtoBS) extruded cell-laden dECM bio-ink at 15 °C, while the printed structures were solidified at 37 °C. To accommodate different stiffness of printed tissues, MtoBS can print 100 μm without the PCL framework for less stiff tissues or 200 μm with the PCL framework for stiffer tissues or organs. Encapsulated human inferior turbinate tissue-derived mesenchymal stromal cells in the 3D printed tissues were able to upregulate the gene expression of cells from which the parental dECM was derived. For example, the heart tissue-derived ECM significantly promoted the expression of cardiogenic proteins Myh6 and Actn1, in comparison to 3D collagen gels. The same group also reported that 3D printed dome-shaped adipose tissue (from decellularized lipoaspirate) were able to differentiate human adipose-derived stem cells into PPAR-γ (peroxisome proliferator-activated receptor-γ) expressing phenotypes in vitro and in vivo [51]. Further, they were able to tailor material properties of dECM bio-ink via vitamin B2-induced UVA crosslinking and thermal gelation to enable printing of CPCs in a construct with biomechanical properties of native cardiac tissue [52].

In sum, 3D bioprinting has emerged from thermal ink-jet printing of single components to the utilization of multiple biomaterials and multiple progenitor and/or stromal cell types with in situ differentiation. Multi-photon imaging-based methods augmented the evolution of 3D bio printing by utilized intact, native ECM and achieving sub-micrometer resolution. Incorporating vasculature has been a design criterion from the beginning to maintain the long-term patency of 3D printed structures both in vivo and in vitro and approaches to meet this criterion are underway. Most recent 3D bioprinting technologies demonstrated feasibility of creating complex anatomical structures based on templates acquired from CT or MR imaging. Finally, digested, dECM has also been used as a component of cell-laden bio-ink to stimulate differentiation of printed stem cells.

#### Comparison of decellularization to 3D bioprinting

Decellularization of tissue and 3D bioprinting of tissue-like constructs have potential to achieve solid organs ex vivo, but each is executed in a completely different fashion. Here, we compare these two technologies such that they might be most appropriately applied to a given tissue-type or regenerative medicine application (Table 1).

**Table 1:** Comparison of features of decellularized tissue vs. 3D bioprinting for solid organ fabrication

Feature	Decellularization	3D Bioprinting
Architecture Fidelity	Retains complex, intact ECM architecture. Retains vascular tree supportive of recellularization.	Attains moderately complex geometries with precision. Simple structures with vasculature have been printed.
Cell Positioning	Precise cell positioning is not possible. Recellularization is perfusion- based and therefore stochastic.	Specific localization of cells at multiple length scales is possible.
Biochemical Signaling	Innate ECM- based biochemical signaling.	Biochemical cues are provided through incorporation of ECM, growth factors or other signaling molecules into the bio-ink.
Mechanical Integrity	Decellularized organs are mechanically weak with limited ability to resist shear; this can improve with recellularization and long term culture.	A range of mechanical properties can be achieved based on bio-ink selection.
Flexibility	Limited availability of donor organs unless xenogeneic tissue is used. Ability to repopulate with multiple cell type is challenging.	Biomaterial selection and design is relatively flexible. However, candidate materials are somewhat limited. Only 2–3 components can be printed simultaneously.
Method Maturity	For certain tissue types optimized procedures have been developed and automated for efficient decellularization and recellularization.	Solid organs with innate vasculature has not yet been realized.
Customization	Customization of size and shape is limited.	Can be tailored for any size or defect.
Immunogenicity	Limited immunogenicity, though studies ongoing with respect to adaptive immune responses.	Largely unexplored, though immunological responses might be avoided by including immunomodulatory agents in bio-ink.
Best Applications	Organs with limited numbers of different cells and high vasculature to tissue ratio (blood vessel, lung, bladder).	Organs with intermediately complex geometries and tight packing (bone, cartilage; more complex geometries such as heart, kidney, liver may be possible in future).

An important attribute for most engineered tissues is the ability to localize cells and matrix with spatial acuity in that augmented functionality is not compromised in the long run. The overall shape and size of a solid organ with decellularization is relatively well conserved in this top down approach (Figs. 1a and 2). Decellularization maintains the natural scaffold with corresponding complex architecture including blood vessels necessary for perfusion-based repopulation. Overall, the structure of the native tissue is more likely to be well-mimicked with decellularization than with 3D bio printing. However cell placement is more challenging with decellularized tissue, because it is dependent on reperfusion and the subsequent ability of cells to attach, move and differentiate with spatial acuity. There is evidence to support this possibility, especially for organs such as the lung with 1) a high vasculature to somatic cell ratio and 2) limited numbers of different cell types. Thus decellularized tissues lack means for scientists to impose spatial control of cell positioning and the outcomes for morphogenesis depend on in situ differentiation of delivered progenitor cells or homing capabilities of differentiated cells. In contrast, 3D bio printing accomplishes spatial acuity in a bottom-up manner (Figs. 1b and 3b). Biop rinting technologies have been unceasingly improved such that biomaterial and cells can be placed at the time of bio printing with high resolution. The ITOP technology is able to print at a few micrometer scales without cells and up to 50 μm with cells. MPE-based fabrication is capable of printing sub-micrometer scale complexities of tissues with biomaterial. In addition, several bio printing modalities can attain organ-level size (Fig. 3b). The FRESH technology demonstrated nearly identical length, width, and size of internal structures with 10 % variability, although the technology was not yet able to print cells within the alginate scaffold. Of note, achieving micrometer-scale resolution and organ-scale size simultaneously is still challenging. At high resolution, the time required to fabricate an organ-scale structure is limiting. In addition, the overall tissue structure may be more crude with 3D bio printing (especially at the current state of technology), but the ability to incorporate not only cells but also signaling molecules that could encourage proper arrangement and function of incorporated cells make this an attractive option for organs that require positioning of multiple cell types with more limited vasculature. Since the purpose of decellularization is to preserve the native ECM proteins, manipulating the ECM proteins from a decellularized organ is rarely pursued except for the use in dECM bio-ink or self-assembling dECM for protein-polymer hybrids. However, 3D bioprinting provides greater flexibility in the selection of biomaterials as long as the materials are printable and easily stabilized. This flexibility offers the opportunity to tailor the bio-ink for appropriate mechanical properties, microstructures, and electromechanical stimulation. Recent examples of bionic composites showed that electromechanical components could be printed within a 3D bio printed organ for augmented functionality [53] or for therapeutic control and electrical stimulation to affect the engineered organs or the host [54, 55]. In addition, patient-specified characteristics of replacement tissues may become important for successful clinical outcomes. Medical imaging can be used to obtain precise spatial information of injured or lost organs, and the shape and size of the targeted organ can be reconstructed via 3D software and then fabricated with 3D bioprinting. This customized approach bypasses the mismatch of size, shape and other physiological conditions between donors and recipients.

Finally, a discussion of immunological outcomes of 3D bio printed or decellularized solid organs for transplantation is warranted. Decellularization chemically, physically and enzymatically depletes immunological components such as α-gal antigen, MHC/HLA (major histocompatibility complex/human leukocyte antigen) and other genetic material (Fig. 1a). Decellularized porcine livers were nonimmunogenic for allogeneic and xenogeneic transplantation over 28 days [56]. Decellularized human hearts after subcutaneous implantation in Sprague-Dawley rats for two weeks contained significantly more CD68^+^/CD163^+^ macrophages [21], suggestive of the presence of immunomodulatory and tissue remodeling macrophage phenotypes in the decellualized ECM [57]. Thus decellularized ECM has been considered immunologically inert, with exhibited constructive remodeling. However, more recent studies reported that adaptive immunity is substantially engaged in response to implanted decellularized ECMs [58]. For example, porcine SIS did not elicit any signs of rejection [59], but did trigger Th2 responses [60]. In addition, human decellularized dermal matrix, AlloDerm®, when implanted into the abdominal wall of adult monkeys, elicited an early and transient antibody response [61]. Given the data associated with immune activation (or lack thereof) are still scattered and somewhat contradictory, continued assessment of short and long term immune responsiveness to decellularized tissue is needed. 3D printing technologies are less mature in assessment of immunologic responsiveness [8, 62]. As these technologies mature, bio-ink selection and design should consider chronic inflammation and adaptive immunity for the transplantation of 3D printed solid organs. Strategic design of bio-ink materials and autologous or HLA-matched allogeneic iPSC [63] may help alleviate potential immunological complications, but this hurdle must be tested for clinical application of solid organs in addition to the viability and lineage-commitment of printed stem or progenitor cells.

## Conclusions

In this review, we have highlighted important findings from the two most promising technologies for solid organ fabrication, decellularized tissue and 3D bio printing. Decellularization began with great promise to regenerate cadaveric organs while overcoming transplant rejection and possibly alleviating perpetual shortage of donated organs. The most recent human decellularized heart was repopulated with RNA-induced PSCs to differentiate cardiomyocytes to avoid the risk of genetic modification of iPSCs. Although the recellularized cardiac slices and fibers maintained beating phenotypes, sarcomeric structures, and electromechanical function, the whole heart scaffold could not exhibit the same extent of functionality that was attained in the cardiac slice and fibers. Thus progress at the organ level scale with decellularized tissue is still in its infancy, but is advancing [14]. 3D bioprinting of tissues, especially of soft tissues is also in its infancy. 3D bioprinting can guide the placement of cell and supporting matrix at levels corresponding to the native tissue. Many 3D bioprinting technologies already showed printed cells maintain good viability. The next step is to create a solid organ with PSCs at an appropriate differentiation stage for transplantation. Further, PSCs or progenitors in bio-inks need to be protected from uncontrolled differentiation during printing and their differentiation, proliferation, and migration should be precisely guided by spatially defined cues from matrices. In vivo or in vitro maturation should be accompanied either by biochemical signaling or electromechanical stimulation [64, 65] to create a functional organ. Despite these challenges, 3D bioprinting technologies discussed here are just beginning to achieve smaller goals for regenerative medicine application including in vitro model systems and drug testing.

4D printing technology emerges as well, conferring printed 3D structures with the ability to change their form or function with time under stimuli such as pressure, temperature, wind, water or light [66, 67]. A candidate material for 4D printing is stimuli-responsive hydrogels mimicking the dynamics of the ECM [68], where the hydrogel material forms a pre-defined 3D configuration. Inspired by nastic plant motion, a calla lily flower was printed and transformed upon swelling (Fig. 3c) [69]. This biomimetic 4D printing technology controls the orientation of cellulose fibrils embedded in a soft acrylamide hydrogel to define elastic and swelling anisotropies. During printing, the composite fibrils undergo shear-induced alignment, leading to printed filaments with anisotropic stiffness and swelling behavior along the filament length. In addition, the anisotropic swelling enables precise control over curvature, which was quantified by a mathematical model for the mechanics of anisotropic objects to manipulate the embedding of a complex surface.

In future, decellularized tissues will continue to find their way to clinical practice as they have already for skin and blood vessel. Generation of certain tissues not amenable to decellularization and more importantly, recellularization, may benefit from current or emerging variations of 3D bio printing.

## Abbreviations

2D, two dimensional; 3D, three dimensional; 4D, four dimensional; ADSC, adipose-derived stem cells; AFSC, amniotic fluid derived stem cell; BSA, bovine serum albumin; CAD, computer aided design; CHO, Chinese hamster ovary; CPC, cardiac progenitor cell; CT, computed tomography; DLP, digital light processing; DMEM, Dulbecco’s Modified Eagle Medium; EC, endothelial cell; ECM, extracellular matrix; ESC, embryonic stem cell; FRESH, freeform reversible embedding of suspended hydrogels; HA, hyaluronic acid; HNDF, human neonatal dermal fibroblast; HUVEC, human umbilical vein endothelial cell; iPSC, induced pluripotent stem cells; ITOP, integrated tissue-organ printer; MCPs, multipotent cardiovascular progenitors; MI, myocardial infarction; MRI, magnetic resonance imaging; PCL, poly(ε-caprolactone); PECAM-1, platelet/endothelial cell adhesion marker-1; PPAR-γ, peroxisome proliferator-activated receptor-γ; SDS, sodium dodecyl sulfate; SEM, scanning electron microscopy; SHG, second harmonic generation; SIS, small intestinal submucosa; SMC, smooth muscle cell; TP-LSL, two-photon laser scanning lithography
